# FSCN1 acts as a promising therapeutic target in the blockade of tumor cell motility: a review of its function, mechanism, and clinical significance

**DOI:** 10.7150/jca.67977

**Published:** 2022-05-09

**Authors:** Zhongxun Li, Jiao Shi, Nannan Zhang, Xiwang Zheng, Yukun Jin, Shuxin Wen, Wanglai Hu, Yongyan Wu, Wei Gao

**Affiliations:** 1 Shanxi Key Laboratory of Otorhinolaryngology Head and Neck Cancer, First Hospital of Shanxi Medical University, Taiyuan 030001, Shanxi, China; 2 Shanxi Province Clinical Medical Research Center for Precision Medicine of Head and Neck Cancer, Department of Otolaryngology Head & Neck Surgery, First Hospital of Shanxi Medical University, Taiyuan 030001, Shanxi, China; 3 Clinical Medical Academy & General Hospital, Shenzhen University, Shenzhen 518055, Guangdong, China; 4 Department of Otolaryngology Head & Neck Surgery, Shanxi Bethune Hospital, Taiyuan 030032, Shanxi, China; 5 Translational Research Institute, People's Hospital of Zhengzhou University, Academy of Medical Science, Henan International Joint Laboratory of Non-coding RNA and Metabolism in Cancer, Zhengzhou University, Zhengzhou 450003, Henan, China

**Keywords:** FSCN1, Actin-binding protein, Epithelial-mesenchymal transition, Metastasis and invasion, Therapeutic target of cancer

## Abstract

Fascin actin-bundling protein 1 (FSCN1) is an actin-bundling protein that is capable of inducing membrane protrusions and plays critical roles in cell migration, motility, adhesion, and other cellular interactions. FSCN1 also plays a role in forming and stabilizing filopodia or microspikes, which assist during cell migration. Furthermore, FSCN1 is a downstream target of several microRNAs and participates in various biological processes, such as epithelial-to-mesenchymal transition and autophagy, which regulate the invasion and migration ability of cells in various cancers. Increased FSCN1 levels have been associated with enhanced migration and invasion of multiple cancers as well as poor patient prognosis. Promising results from* in vitro* experimental studies using docosahexaenoic acid (DHA) in breast cancer and recombinant porcine NK-lysin A in hepatocellular carcinoma have revealed that anticancer drugs targeting FSCN1 have significant potential clinical applications. This review discusses FSCN1 in terms of five aspects: structure and function, biological processes, regulatory mechanisms, clinical applications, and future prospects.

## Introduction

Fascin actin-bundling protein 1 (FSCN1), also called fascin or fascin-1, is a globular, filamentous, actin-binding protein, that belongs to the actin cytoskeletal protein family 1. Through stabilization of actin bundles, FSCN1 supports various cell structures, including microspikes, filopodia, lamellar pseudopods, and other actin-based protrusions under the plasma membrane, which are important for processes including cell migration and cell matrix adhesion 2, 3. Recently, FSCN1 has attracted much attention, because multiple studies have indicated that it may be a candidate biomarker or therapeutic target for the treatment of several different aggressive, metastatic carcinomas 2, 4, 5. In this review, we discuss, at length, the structure and biological function of FSCN1, and describe the expression patterns, biological processes, and regulatory mechanisms of FSCN1 in various cancers. Furthermore, we discuss the clinical significance of FSCN1 in various cancers.

## FSCN1 gene and protein structures

**Figure 1:**
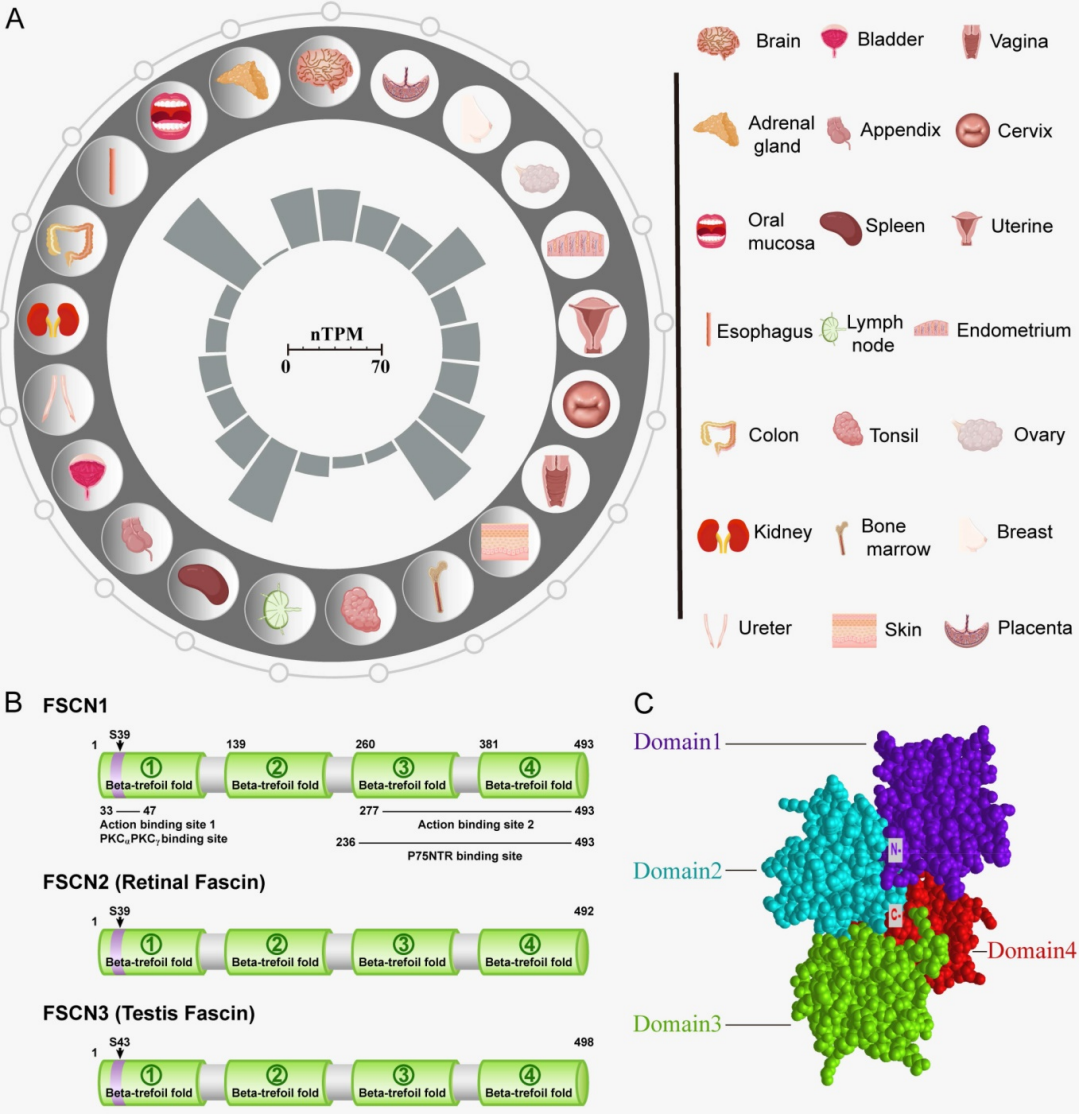
**Structure and biological functions of FSCN1.** A: Expression of FSCN1 in normal human organ tissues. The inner barchart on the left panel shows the expression profile of FSCN1 in normal human organs. The normalized expression (nTPM) values were obtained from The Human Protein Atlas; The right panel shows the name of the corresponding organ in the left panel. B: Schematic diagram of model proteins in three vertebrates; C: The three-dimensional structure of human FSCN1 protein; “N-” represents the N-terminal of FSCN1; “C-” represents the C-terminal of FSCN1.

In vertebrates, the expansion of the FSCN family has proceeded through a unique sequence of gene duplications. Three forms of FSCN exist in vertebrates, namely FSCN1, FSCN2, and FSCN3. FSCN1 is widely expressed in the nervous system as well as mesenchymal tissues (Figure 1A). FSCN2 is expressed in retinal photoreceptor cells, whereas FSCN3 is only expressed in the testis 2, 6. In humans, *FSCN1* is located on chromosome 7p22 7, and *FSCN2* is located on chromosome 17q25. In addition, each gene is adjacent to a member of the actin gene family 8, 9. *FSCN1* and *FSCN2* share a 56% sequence identity, whereas* FSCN3* exhibits 27% sequence identityhomology to *FSCN1* and 28% to *FSCN2* (Figure 1B) 4.

The FSCN1 protein comprises four β-trefoil domains (Domain 1: residues 1-139, Domain 2: residues 140-259, Domain 3: residues 260-382, and Domain 4: residues 383-493) (Figure 1C) 1. Evidence suggests that Ser39 and Ser274 are related to phosphorylation-dependent regulation of actin-binding in FSCN1 10, 11. In addition, phosphorylation of FSCN1 at tyrosine 23 and Ser 38 is important for cell migration and filopodia formation in esophageal squamous cancer cells 12. Monoubiquitination is a type of post-translational modification that regulates the actin-bundling activity and dynamics of FSCN1. FSCN1 is monoubiquitinated at two lysine residues (Lys247 and Lys250) in Domain 2 13. FSCN1 plays a role in tissues through two types of actin-based structures, namely, dynamic cortical cell protrusions and cytoplasmic microfilament bundles. The cortical structures include filopodia, spikes, lamellipodial ribs, oocyte microvilli, and the dendrites on dendritic cells, which play roles in cell-matrix adhesion, cell interactions, and cell migration. In contrast, the cytoplasmic actin bundles appear to be involved in cell architecture. The main function of FSCN1 is crosslinking of actin microfilaments into tight, rigid, and parallel bundles 14.

## FSCN1 expression patterns and distribution in various cells and tissues

FSCN1 expression patterns exhibit spatiotemporal specificity. In *Drosophila*, FSCN1 is expressed in numerous cell types throughout the life cycle. In mouse embryos, *Fscn1* is expressed in the nervous system (brain, spinal cord, and eye), developing somites, the condensing mesenchyme of limb buds, the skeletal and smooth muscle of various organs, and in heart ventricles at low levels 15. The expression pattern of *FSCN1* in human embryos and tissues is similar to that observed in mouse embryos 16. Knockout of *FSCN1* in an inbred strain of mice resulted in about 48% neonatal lethality, and the bodyweight of the surviving mice in the strain was observed to be reduced 17. FSCN1 also plays a role in the formation and stability of filopodia or microspikes, which assist in cell migration 17-19. The contractile phenotype of vascular smooth muscle cells (VSMCs) is converted to a migratory phenotype upon blood vessel injury, and FSCN1 is an important component of the dynamic podosomes that mediate VSMC migration in response to PDGF receptor signaling through c-Src kinase 20. FSCN1 also plays a critical role in the formation of specialized podosomes, known as invadopodia, which mediate the invasion of melanoma-derived cells into a three-dimensional extracellular matrix 21. This process is closely related to the invasion and migration capabilities and processes of cancer cells.

## Biological processes involving FSCN1

### Elevated FSCN1 levels promote epithelial-to-mesenchymal transition (EMT)

EMT is a dedifferentiation process that converts adherent epithelial cells into singular migrating cells, which are essential for embryonic development, oncogenic progression, and metastasis 22. EMT promotes stemness in normal breast tissues and breast cancer cells 22. In addition, embryonic stem cell genes, including *Oct4* and *Nanog*, positively regulate tumor metastasis through the enhancement of EMT in lung adenocarcinoma cells 23. FSCN1 is a downstream effector of SNAI2 in the promotion of EMT, and FSCN1 mRNA levels are significantly elevated after the induction of transforming growth factor β (TGF-β) expression 24. FSCN1 regulates EMT in various cancers, including ovarian cancer 25, squamous cell carcinoma, and lung cancer 26, 27. Moreover, data obtained from 80 samples of gastric cancer patients indicated that FSCN1 promotes EMT in gastric cancer 28. EMT is also a primary mechanism that contributes to resistance to chemotherapy in hepatocellular carcinoma 29.

**Figure 2:**
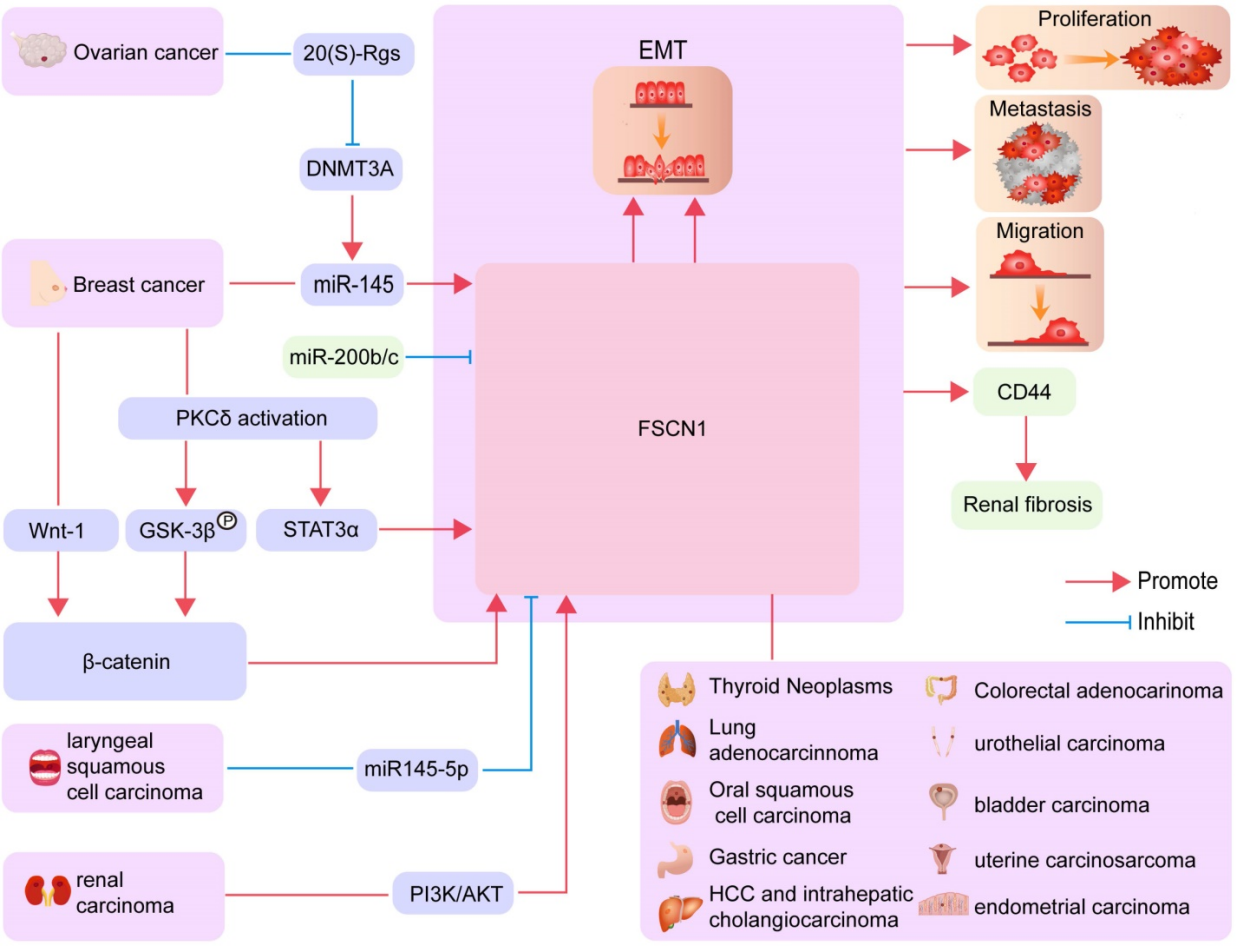
** The biological processes involving FSCN1 in different cancers.** FSCN1 mediated EMT, proliferation, metastasis, and migration in various cancers are shown.

The microRNA (MiR)-200b/c family is known to regulate the EMT process through directly targeting the FSCN1/CD44 axis and thereby inhibiting renal fibrosis 30. A recent study reported that the suppression of miR‑145 expression in breast cancer cells affects cell migration by targeting FSCN1 and inhibiting EMT 31. Inhibition of FSCN1 significantly suppresses vimentin expression and increases E-cadherin expression. Therefore, increased FSCN1 expression levels may promote the resistance of HCC against DOX by inducing EMT 32. Research in the field of ovarian cancer has revealed that 20-(S)-Rg3 prevents EMT by targeting the DNMT3A/miR-145/FSCN1 pathway (Figure 2) 25.

### Increased FSCN1 expression inhibits autophagy

Autophagy is a mechanism involving self-digestion in eukaryotic cells that removes abnormal proteins and damaged organelles through lysosomal degradation 33. The role of autophagy in tumor biology is relatively complex 34. During the early stages of tumor development, inhibition of autophagic activity induces continuous precancerous cell growth 35. However, autophagy also enables the survival of advanced tumor cells in conditions of nutrient-limitation and low oxygen 36, 37. In the endometrium, elevated FSCN1 levels reverse the inhibitory effect of autophagy on cell invasion. Additionally, the activation of autophagy inhibits the formation of filopodia by FSCN1 (Figure 2) 38, 39.

## Biological functions and molecular mechanisms of FSCN1 in cancer Cancers of the nervous system

FSCN1 is expressed in cells of the central nervous system, such as microglia, astrocytes, and neurons, and is present in glioblastomas of all grades 40, wherein its function appears to be associated with cell motility, invasion, and immune response 41, 42. FSCN1-mediated formation of filopodia is essential for mouse development 17. In rat spinal cords, FSCN1 contributes to neuropathic pain through the promotion of inflammation 43. FSCN1 also regulates the migration of subventricular zone-derived neuroblasts in the postnatal brain 44. Furthermore, there is a correlation between increased FSCN1 expression and increasing grades of astrocytomas 45. FSCN1 is also one of four proteins that are downregulated in the thalamus of stargazer mutant mice after γ-butyrolactone-induced seizures 46. Additionally, increased FSCN1 expression has been observed in the hippocampus of Down's syndrome poly transgenic mice exhibiting impaired learning and memory 47.

### Malignancy of the lymphatic system

Multiple cell types within the cardiovascular system express FSCN1, including dendritic cells, B lymphocytes, T lymphocytes, macrophages, neutrophils, platelets, vessel wall endothelial cells, smooth muscle cells, and fibroblasts 48. In normal human peripheral blood, FSCN1 expression is restricted to dendritic cells, which play a primary role in the initiation of acquired immune responses (Figure 2) 49-51.

In a previous study, 187 patients with Hodgkin's disease, including 132 patients with nodular sclerosis, 34 with mixed cellularity, 14 with lymphocyte predominance (nodular), 2 with lymphocyte depleted, and 5 with unclassified types were evaluated. In all patients, except those with nodular lymphocyte predominance type, Reed-Sternberg cells and variants were found to be uniformly reactive for FSCN1. In most cases, almost all Reed-Sternberg cells and variants exhibited strong diffuse cytoplasmic staining. In some cases, the intensity of staining was varied. In the nodular sclerosis type, reactive cells frequently appeared in the form of aggregates, sheets, or syncytial masses mixed with recognizable interdigitating reticulum cells. These results indicated that FSCN1 represents a highly effective marker for the detection of specific dendritic cells in normal and neoplastic tissues and is a highly consistent marker for Reed-Sternberg cells and variants of the Hodgkin's disease 50.

### Head and neck cancers

Laryngeal squamous cell carcinoma (LSCC) is a common form of head and neck cancer and is generally associated with a poor prognosis. Quantitative RT-PCR and western blot analyses revealed that FSCN1 expression is significantly upregulated in LSCC tissues compared with that in adjacent normal mucosa tissues 52. Loss-of-function studies showed that FSCN1 knockdown inhibited LSCC migration, invasion, and growth through the suppression of EMT 53. Furthermore, Gao et al. identified that FSCN1 binds with AIMP1 and LTA4H in LSCC, and their results suggested that AIMP1 and LTA4H were possible effectors involved in FSCN1-mediated malignant progression of LSCC 54. In addition, expression of FSCN1 is higher in tongue squamous cell carcinoma (TSCC) tissues and cells than in adjacent non-carcinoma tissues and normal control cells. Knockdown of *FSCN1* inhibited TSCC cell viability and trans-migration *in vitro* and impaired tumor growth *in vivo* 55.

### Cancers of the respiratory system

In the lungs, FSCN1 is directly recruited to mitochondria under metabolic stress conditions to stabilize mitochondrial actin filaments, thereby promoting mitochondrial oxidative phosphorylation by increasing the biogenesis of respiratory complex I. Similarly, FSCN1 promotes the metastatic colonization of lung cancer cells by enhancing metabolic stress resistance and mitochondrial oxidative phosphorylation 56. Furthermore, FSCN1 is differentially expressed in non-small cell lung cancer (NSCLC) tissues and normal para-carcinoma tissues; moreover, FSCN1 expression in cancer tissues is associated with poor prognosis in patients with NSCLC (Figure 2) 57.

### Cancers of the digestive system

Oral squamous cell carcinoma exhibits aggressive progression with a high incidence of nodal metastasis, even in the early stage 58. In 2007, it was first demonstrated that FSCN1 overexpression was significantly associated with lymph node metastasis and tumor recurrence but not tumor stage or differentiation in oral squamous cell carcinoma (Figure 2) 59.

FSCN1 expression levels in esophageal squamous cell carcinoma are often increased compared with those in the normal epithelium, and FSCN1 overexpression is significantly associated with poor prognoses 60, 61. Furthermore, non-phosphorylation mutations at tyrosine 23, serine 38, and serine 39 in β-trefoil domain 1 and at serine 274 in β-trefoil domain 3 promote cell motility and filopodia formation, while phosphorylation mutations at these sites inhibit cell functions and filopodia formation 12.

Normal gastric epithelial cells are negative for FSCN1 expression, whereas endothelial cells, lymphocytes, and stromal cells in the underlying lamina propria are positive. FSCN1 is mainly located in the cytoplasm of gastric adenocarcinoma cells (Figure 2) 62. Enhanced immunostaining intensity of FSCN1 is correlated with higher histological grades, AJCC staging, and worse prognoses in Chinese patients with gastric adenocarcinoma 63.

In HCC, FSCN1-positive tumors are larger and less differentiated than are FSCN1-negative tumors, and they are more prone to portal venous invasion, bile duct invasion, and intrahepatic metastasis. In intrahepatic cholangiocarcinoma, FSCN1 exhibits similar expression characteristics (Figure 2) 64. Additionally, FSCN1 expression is significantly correlated with high alpha-fetoprotein levels in HCC. Patients with FSCN1-positive HCC exhibit significantly poorer outcomes than patients with FSCN1-negative HCC, and FSCN1 is an independent prognostic factor for disease-free survival of HCC patients 65. Through global gene expression analysis, FSCN1 levels were found to be increased during the transition from carcinoma *in situ* to invasive adenocarcinoma 66.

FSCN1 expression is downregulated in normal colonic epithelial cells. However, FSCN1 is expressed in subsets of adenomas and colorectal adenocarcinomas. Specifically, FSCN1 is widely expressed in 16% of adenomas and between 17% and 26% of adenocarcinomas. In 47% of colorectal tumors, FSCN1 expression is increased in the surrounding stroma regardless of the level of FSCN1 in the tumor. In adenomas, FSCN1 and Ki67 expressions are often inversely correlated at the cellular level, but this trend is less evident in adenocarcinomas. In advanced tumors, strong FSCN1 staining is significantly associated with poor prognosis. FSCN1 regulates cell morphology and migration and may represent a potential, novel marker or therapeutic target for the identification and treatment of patients with aggressive forms of colorectal adenocarcinoma (Figure 2) 67.

### Cancers of the urinary system

FSCN1 expression is increased in actively growing renal carcinoma cell lines 7 compared with that in normal kidney cells 68. Differences in the extent and intensity of FSCN1 immunohistochemical staining are useful to predict patient prognosis 69. Similarly, increased FSCN1 levels are associated with aggressiveness of renal cell carcinoma in patients 70. Additionally, increasing evidence suggests that FSCN1 is an effective predictive factor of tumor clinicopathological parameters in renal cell carcinoma 71. In renal carcinoma cell lines, treatment with a PI3K or AKT inhibitor reduces FSCN1 protein and mRNA expression, indicating that FSCN1 may be regulated through the PI3K/AKT axis (Figure 2) 72.

In urothelial carcinoma tissues, increased FSCN1 expression is positively correlated with histological grade and pT stage. FSCN1 is also positively correlated with increased migration and invasion of cancer cells 73. Furthermore, FSCN1 overexpression is associated with increased invasiveness of carcinomas in the urinary bladder (Figure 2) 74.

### Gynecologic Cancer

FSCN1 directly affects the constitutive expression of the downstream targets of β-catenin and enhances the self-renewal ability. It is also crucial for the expression and function of the downstream targets of β-catenin induced by glycogen synthase kinase 3b inhibition. Moreover, the constitutive and inducible expression of the downstream targets of β-catenin mediated by FSCN1 depends, at least in part, on focal adhesion kinase (FAK). Among breast cancer patients, those with co-expression of FSCN1^high^ and FAK^high^ or high β-catenin downstream targets exhibit the least favorable survival outcomes, whereas in the FSCN1^low^ group, the co-expression of FAK^high^ or high β-catenin downstream targets has a less significant effect on patient survival 75.

In uterine carcinosarcoma, FSCN1 is abnormally expressed and is associated with aggressive metastatic behavior and poor prognoses 76. Moreover, increased FSCN1 levels contribute to the highly invasive properties of endometrial carcinoma and can predict an epithelial-mesenchymal transition-like process. FSCN1 overexpression in intravascular tumor cells indicated increased metastatic risk, suggesting that FSCN1 may be an independent prognostic indicator for the different steps of extracellular matrix invasion 77-79.

## The mechanisms underlying aberrant FSCN1 expression in cancer

### Hypoxia-inducible factor 1 promotes FSCN1 expression

**Figure 3:**
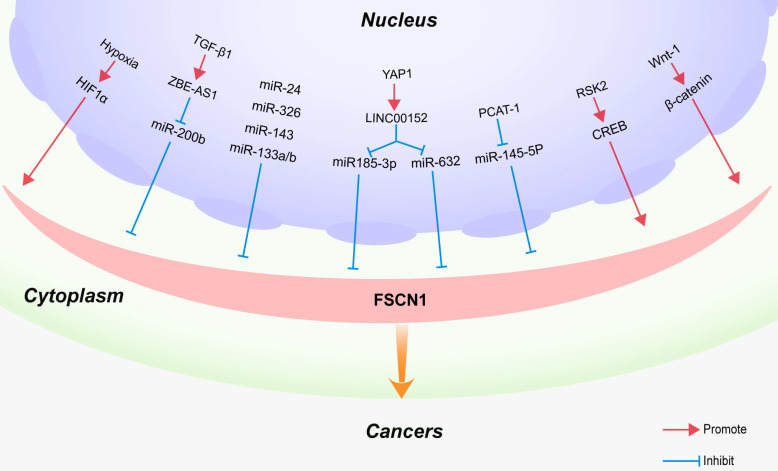
** Regulatory mechanism of FSCN1 in cancers**. Regulatory factors or signaling pathways that affect FSCN1 expression in various cancers are shown.

Hypoxia is one of the most common types of microenvironmental stress conditions observed in solid tumors. It is the result of excessive tumor growth and insufficient blood supply, which play a central role in tumor metastasis 80-82. Hypoxia‑inducible factor‑1 (HIF‑1) is an important heterodimeric transcription factor comprising a highly regulated α subunit and a constitutively expressed β subunit. HIF‑1α is a key mediator of cellular responses to hypoxia and plays a critical role in regulating HIF‑1 transcriptional activity 83. Additionally, it regulates the expression of target genes related to tumor invasion and metastasis 84. HIF‑1α plays an important role in the tumor invasion and metastasis of head and neck squamous cell carcinoma 85. FSCN1 is a direct target of HIF‑1α, and HIF‑1α may promote invasion and metastasis by upregulating the expression of FSCN1 in hypopharyngeal squamous cell carcinoma and pancreatic ductal adenocarcinoma (Figure 3) 86.

### Long non-coding RNAs regulate FSCN1 expression

Long non-coding RNAs (lncRNAs) are a class of regulatory transcripts, longer than 200 nucleotides, with no protein-coding potential. lncRNAs act as molecular sponges for microRNAs, thereby reducing their influence on target mRNAs. The lncRNA ZEB1 anti-sense RNA 1 (ZEB1-AS1) functions as an oncogenic lncRNA in many types of cancers 87. In recent studies, ZEB1-AS1 was identified as a downstream target of TGF-β1 and reported to contribute to the TGF-β1-mediated regulation of cell migration and invasion by upregulating the expression of the FSCN1 axis in bladder cancer cells (Figure 3) 88.

As the main effector of the Hippo pathway, Yes-associated protein 1 (YAP1) plays a key role in the regulation of a variety of biological functions, including intercellular contact inhibition, proliferation, and differentiation 89, 90. YAP1 is a transcription coactivator that is highly expressed in colorectal cancer (CRC) 91-93. Recently, a novel YAP1 regulatory model was proposed, which has attracted widespread attention. In this model, YAP1 transcriptionally regulates noncoding RNAs (ncRNAs) in CRC, including microRNAs such as miR-130a 93, miR-29 94 and lncRNAs such as RMRP 95, BCAR4 96, MALAT1 97, and lncARSR 98. Among these lncRNAs, LINC00152 is expressed at high levels in human CRC tissues. The suppression of LINC00152 expression results in the downregulation of 159 genes, thereby resulting in the inhibition of the malignant proliferation, invasion, and metastasis of CRC cells. LINC00152, as a novel YAP1 target, promotes the biological characteristics of CRC cells by sponging miR-185-3p and miR-632 to upregulate FSCN1 expression. These results suggest that the YAP1/LINC00152/FSCN1 axis promotes the malignant proliferation, migration, and metastasis of CRC (Figure 3) 99.

Bioinformatics analyses revealed that the seed regions of PCAT-1 and miR-145-5p exhibit some complementary pairing 100. MiR-145-5p has been reported to exhibit an anti-oncogenic function in various cancers, including laryngeal cancer, gastric cancer, breast cancer, and renal cell carcinoma 101-103. Moreover, miR-145 inhibits cell proliferation, migration, and invasion by targeting FSCN1 in prostate cancer 104. Recent studies indicate that PCAT-1 acts as a competing endogenous RNA of miR-145-5p to upregulate FSCN1 expression, thereby promoting the development of prostate cancer (Figure 3) 100.

### MicroRNAs regulate FSCN1 expression

MicroRNAs (miRNAs) are non-coding RNAs that have a length of 18-25 nucleotides. miRNAs suppress gene expression at the post-transcriptional level by binding to the 3'‑untranslated region of mRNAs 105. MiR-133a negatively regulates various types of human malignant cancer cells, including NSCLC 106, gastric cancer 101, osteosarcoma 107, esophageal squamous cell carcinoma 108, ovarian cancer 109, and colorectal cancer 110. By inhibiting the protein expression of FSCN1, miR‑133a partially inhibits the invasion of colorectal cancer cells (Figure 3) 111. Wu et al. revealed that miR-488 inhibits the proliferation and motility of breast cancer cells by downregulating FSCN1 expression 112. Furthermore, the FSCN1 gene has been identified as a direct target of several miRs, such as miR-145-5p in laryngeal cancer 53, miR-143 in chondrosarcoma and esophageal carcinoma 113, miR-24 in nasopharyngeal and prostate cancers 114, 115, and miR-326 in lung and gastric cancers (Figure 3) 116.

### CREB signaling upregulates FSCN1 expression

cAMP response element-binding protein (CREB) and CREB-binding protein (CBP)/p300 play critical roles in epithelial tumorigenesis 117. CREB is a transcription factor, whose signaling is implicated in the promotion of tumor progression, growth stimulation, apoptotic resistance, and the support of angiogenesis 118, 119. Li et al. reported that activation of the CREB signaling pathway upregulates FSCN1 expression in breast adenocarcinoma, head and neck squamous cell carcinoma, and lung adenocarcinoma cells, leading to enhanced cancer cell invasion *in vitro* and tumor metastasis *in vivo* 120. In neuronal precursor cells, knockdown of CBP downregulates mRNA and protein expression levels of FSCN1, indicating that FSCN1 is a downstream target of CBP 121.

### Activation of the Wnt/βcatenin signaling pathway upregulates FSCN1 expression

The canonical Wnt/β-catenin signaling pathway has multiple functions in epithelial cancers, and increased levels of β-catenin accumulate in the nucleus during the late stages of tumor progression. β-catenin accumulation in the nucleus leads to its interaction with TCF/LEF factors, driving the transcription of target genes 122. It has been demonstrated that the *FSCN1* gene is a direct downstream target of the Wnt/β-catenin signaling pathway in colorectal cancer cells. Immunohistochemical staining revealed that FSCN1 was specifically localized at the invasive front of colon carcinomas that display nuclear β-catenin 123. Consistent with these findings, Kim et al. demonstrated that inhibition of the Wnt/β-catenin pathway through the silencing of galectin-3 reduced FSCN1 expression in gastric cancer cells 124. Furthermore, Hölsken et al. reported that knockdown of β-catenin through RNA interference significantly downregulated FSCN1 mRNA expression levels in adamantinomatous craniopharyngioma cells (Figure 2) 125.

## Clinical significance of FSCN1 in cancer treatment

Owing to the extensive investigations into the functions of FSCN1, which have led to a greater understanding of its biological functions and mechanisms, FSCN1 is increasingly being used as a therapeutic target in cancer treatment. As a biological marker for EMT, FSCN1 is more sensitive and specific than the current standards that are used to diagnose antibody-mediated rejection, and it also exhibits good prognostic value for predicting future graft dysfunction 126. Knockdown of the lncRNA CCAT1 enhances paclitaxel sensitivity in prostate cancer by regulating the expression of miR-24-3p and FSCN1 114. Additionally, FSCN1 knockdown inhibits cell migration and invasion in NSCLC through the regulation of the MAPK signaling pathway 127. Co-targeting the epidermal growth factor receptor and FSCN1 has been proposed as a novel treatment strategy for the treatment of triple-negative breast cancer 128. Furthermore, FSCN1-targeting anti-tumor drugs have been used in the treatment of various cancers. Temozolomide induces the expression of big potassium ion channels and inhibits FSCN1 expression in glioma, indicating that FSCN1 is a possible target for the treatment of glioma 129. Docosahexaenoic acid inhibits breast cancer cell migration through the inhibition of FSCN1 130. Recombinant porcine NK-lysin inhibits the invasion of HCC *in vitro*, suggesting that this compound may be a potential therapeutic candidate that can be used in HCC treatment 131.

FSCN1 is a key downstream component of several crucial pathways associated with tumor progression, including EMT, the Wnt/β-catenin signaling pathway, and the expression of microRNAs. FSCN1 expression can help predict poor prognosis in patients with nasopharyngeal carcinoma 132. Remarkably, several recent studies have highlighted the potential of FSCN1 as a novel prognostic biomarker and therapeutic target for the treatment of a variety of cancers [Table 1].

The inhibition of FSCN1 can lead to the reduction of invasion and migration capabilities of cells in a variety of cancers. The success of *in vitro* experimental studies regarding the role of DHA in breast cancer and that of recombinant porcine NK-lysin A in HCC have demonstrated that anticancer drugs targeting FSCN1 have significant potential for clinical applications 130, 131. Co-targeting the epidermal growth factor receptor and FSCN1 has proved to be a promising novel therapeutic strategy for the treatment of triple-negative breast cancer 128. With the advent of molecular targeted drugs, FSCN1 may have the potential to become a key target in cancer treatment. Improving our understanding of the mechanisms by which FSCN1 promotes cancer cell migration and invasion is therefore an essential initial step toward the development of FSCN1-specific drugs for cancer therapy in the future.

## Figures and Tables

**Table 1:** Role of FSCN1 in the diagnosis and treatment of different cancers

Tumor Type	Related Drugs	Diagnosis	Treatment	References
Adrenocortical Carcinoma	None	poor prognostic markers	a potential therapeutic target	135, 136
Advanced Breast Cancer	None	poor prognostic markers	/	137
Bladder Urothelial Carcinoma	None	poor prognostic markers	a potential therapeutic target	74, 138, 139
Borderline Ovarian Tumor	None	poor prognostic markers	/	140
Breast Cancer	None	None	a potential therapeutic target	128, 141-144
Cholangiocarcinoma	None	poor prognostic markers	a potential therapeutic target	145, 146
Colorectal Cancer	None	poor prognostic markers	a potential therapeutic target	147-149
Esophageal Carcinoma	None	poor prognostic markers	a potential therapeutic target	150-154
Gastric Cancer	None	None	a potential therapeutic target	155
Hepatocellular Carcinoma	Doxycycline, Recombinant Porcine NK-lysin	poor prognostic markers	a potential therapeutic target	32, 65, 131, 156, 157
Laryngeal Squamous Cell Carcinoma	None	poor prognostic markers	a potential therapeutic target	52-54, 134
Lung Cancer	None	poor prognostic markers	a potential therapeutic target	57, 158-160
Nasopharyngeal Carcinoma	Doxorubicin	None	a potential therapeutic target	161
Oral Squamous Cell Carcinoma.	None	poor prognostic markers	a potential therapeutic target	162, 163
Ovarian Cancer	None	None	a potential therapeutic target	164-167
Pancreatic Cancer	None	poor prognostic markers	a potential therapeutic target	66, 86, 168-171
Renal Cell Carcinoma	None	poor prognostic markers	/	70, 172
Thyroid Neoplasms	None	poor prognostic markers	/	173

## Abbreviations

FSCN1: Fascin actin-bundling protein 1
LSCC: laryngeal squamous cell carcinoma
CBP: CREB-binding protein
CRC: colorectal cancer
CREB: cAMP response element-binding protein
DLD: dihydrolipoamide dehydrogenase
EMT: epithelial-to-mesenchymal transition
ENO2: enolase 2
HCC: hepatocellular carcinoma
HIF‑1: hypoxia‑inducible factor‑1
lncRNA: long non-coding RNA
miR: microRNA
miRs: microRNAs
mRNAs: messenger RNAs
NSCLC: non-small cell lung cancer
PCAT-1: prostate cancer-related lncRNA transcript 1
TGF: transforming growth factor
VSMC: vascular smooth muscle cells
ZEB1: zinc finger E-box binding homeobox 1
ZEB1-AS1: ZEB antisense RNA 1
